# When to think about a *Lachesis muta* envenomation in the Western Brazilian Amazon: Lessons from a case report

**DOI:** 10.1590/0037-8682-0027-2022

**Published:** 2022-09-19

**Authors:** Jacqueline de Almeida Gonçalves Sachett, Ana Paula Saboia Marinho, Marizete Melo de Oliveira Santos, Hui Wen Fan, Paulo Sérgio Bernarde, Wuelton Marcelo Monteiro

**Affiliations:** 1 Universidade do Estado do Amazonas, Manaus, AM, Brasil.; 2 Fundação de Medicina Tropical Doutor Heitor Vieira Dourado, Manaus, AM, Brasil.; 3 Hospital das Clínicas Raimundo Chaar, Brasileia, AC, Brasil.; 4 Instituto Butantan, São Paulo, SP, Brasil.; 5 Universidade Federal do Acre, Campus Floresta, Laboratório de Herpetologia, Cruzeiro do Sul, AC, Brasil.

**Keywords:** Snakebite, Lachesis, Antivenom

## Abstract

In the Brazilian Amazon, envenomations by lancehead pit vipers prevail across the region, while bushmaster (*Lachesis muta)* envenomations are rarely confirmed. Here, we described a moderate snakebite, diagnosed as a lancehead pit viper envenomation upon admission and treated with four vials of *Bothrops* antivenom. Blood remained unclottable for 4 days of hospitalization. On day 4, after admission, the patient presented pictures of the perpetrating snake to the hospital staff, which was identified as a *Lachesis muta* specimen. After administering 10 vials of *Lachesis* antivenom, blood became clottable 12 hours after treatment. The patient was discharged without complaints.

## INTRODUCTION

The bushmaster snake, *Lachesis muta* (Linnaeus 1766), is the largest venomous snake in South America, reaching more than 3 meters in length, and is popularly known as “pico-de-jaca” in Brazil^1^. This species occurs in Colombia, Ecuador, Brazil, Venezuela, Suriname, French Guiana, Guyana, Trinidad, Peru, and Bolivia^2^. In Brazil, it is widespread in the Amazon and northern Atlantic Forest (from Ceará to Rio de Janeiro states) biomes, forming a disjunct distribution with marginal records in the northern portion of the Cerrado and a single record in the upland forest in the Caatinga biome^3^. The bushmaster is a snake found mainly in upland forests, though it can also be seen in cocoa plantations and occasionally in pasture areas adjacent to forests^1^ ^,^ ^4^.

Throughout its geographic distribution, it is infrequently registered in studies, probably because it occurs in low population density^1^ ^,^ ^4^. The rarity of this species must also reflect the rarity of confirmed bites by this snake^5^ ^,^ ^6^ and the few published reports of envenomations that occur in nature^7^. In Brazil, clinical reports of envenomations by *L. muta* involve cases that occurred in nature in the central Amazon and Atlantic Forest in the northeastern region of the country^7^, in addition to four cases involving specimens reared in captivity^8^.

Confusion between *Bothrops* and *Lachesis* envenomations is common in the Amazon, which leads to misdiagnosis due to similar clinical signs and symptoms, and generally leads to an over-reporting of *Lachesis* cases^1^. In this study, we describe a case of persistent coagulopathy after *Bothrops* antivenom treatment and late diagnosis of a *Lachesis* envenomation with the correct case management that was subsequently applied. 

## CASE REPORT

**FIGURE 1:**
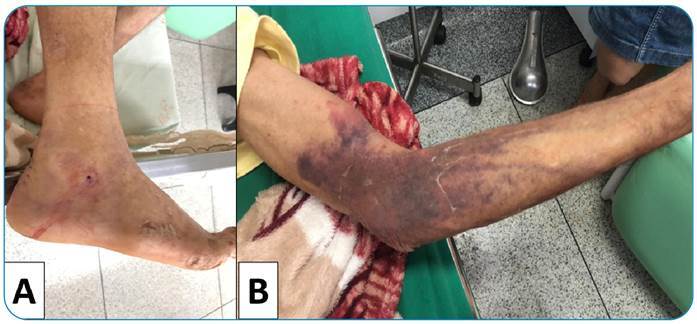
**A)** Bite site on the outer side of the right foot upon admission, and **B)** extensive ecchymosis in the right arm 15 hours after admission.

A 75-year-old male from the Brasileia municipality, state of Acre, in the Western Brazilian Amazon, was admitted to the local hospital after a snakebite to the outer side of the right foot, near the ankle. The snakebite occurred on July 6, 2020, at approximately 3 p.m. while he was working in the woods. The patient reported immediate pain at the site of the bite. Upon arrival at his home, after 10 minutes of walking, the family took him to the hospital. The patient arrived at the hospital approximately 45 minutes after the bite, presenting a punctiform mark with bleeding at the site and mild edema in the area (Figure 1A). In addition, he reported local pain and epigastric pain. Physical examination revealed psychomotor agitation, arterial hypotension (59 x 39 mmHg), tachycardia (103 bpm), sudoresis, an axillary temperature of 35 ºC, and 86% oxygen saturation. Blood was unclottable using the Lee-White clotting test. The patient was diagnosed with *Bothrops* envenomation and treated with four vials (40 mL in total) of *Bothrops* antivenom. In addition, he received intravenous saline and was prescribed 1 g of tenoxicam every 12 hours (EV), 40 mg of omeprazole (EV), 250 mg of hydrocortisone (EV), and a single dose of 50 mg of tramadol (EV).

Three hours after hospital admission, patient examination showed an arterial pressure of 100 x 57 mmHg, heart rate of 70 bpm, and 98% oxygen saturation. Blood remained unclottable, and two more vials of *Bothrops* antivenom (20 mL in total) were administered. Ceftriaxone at 1 g every 12 hours (EV) was also prescribed. 

Nine hours after hospital admission, the patient’s blood was still unclottable, and two more vials of *Bothrops* antivenom (20 mL) were administered. The patient presented an arterial pressure of 88 x 59 mmHg, a heart rate of 58 bpm, and 97% oxygen saturation.

Fifteen hours after hospital admission, the patient reported improvement in pain levels and was hemodynamically stable. However, an extensive ecchymosis appeared in the upper limbs, mainly in the areas close to the venous access (Figure 1B).

On day 3, the patient’s blood was still unclottable blood, and 4 more vials of *Bothrops* antivenom were administered, making a total of 12 vials, which corresponds to the dosage used for severe *Bothrops* envenomations.

**FIGURE 2:**
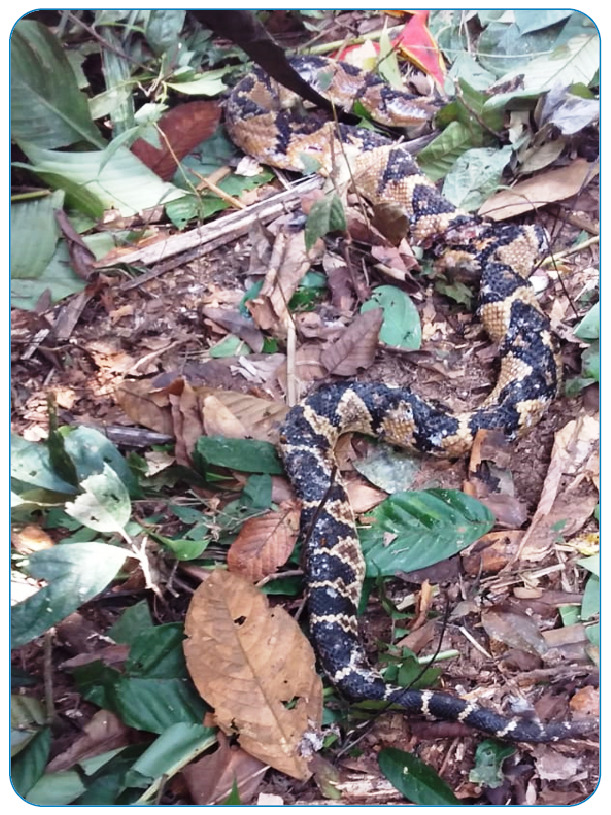
Snake responsible for the envenomation.

On day 4, blood was still unclottable, but no new sites of ecchymosis or active bleeding were observed. The patient had a slightly distended abdomen, an abdominal ultrasound exam was performed, and mild ascites was identified. On this day, the patient got a family member to take a picture of the perpetrating snake, which he had killed after the bite. A herpetologist was consulted, and the snake was identified as *L. muta* (Figure 2). A total of 11 vials of *Lachesis* antivenom (110 mL) were then administered. 

On day 5, the abdomen was globose with abdominal pain identified by superficial and deep palpation. Simethicone was prescribed. Clotting time was normal on this day. 

Finally, on day 6, blood was clottable, but he had constipation, abdominal pain, decreased air-borne sounds, and diffuse pain on deep palpation. Simethicone was continued, and mineral oil was prescribed. An enema was given, which resulted in an abundant evacuation. At the end of the same day, the patient was discharged.

Table 1 presents the clinical and laboratory parameters during hospital follow-up.

**TABLE 1:** Clinical and laboratory parameters during hospitalization.

Parameters	06/07 (Day 1)	07/07 (Day 2)	08/07 (Day 3)	09/07 (Day 4)	10/07 (Day 5)
Hemoglobin (g/dL)	14.9	12.6	11.8	11.7	
Hematocrit (%)	43.6	36.9	34.5	34.1	
Leucocytes (/mm³)	16.9	11.1	11.1	10.9	
Neutrophils (%)	76	80	84	87	
Lymphocytes (%)	21	15	13	10	
Platelets x 10^3^ (/µL)	274	188	188	185	
LW Clotting test	Unclottable	Unclottable	Unclottable	Unclottable	Clottable
Urea (mg/dL)		49			
Creatinine (mg/dL)		0.9			
Aspartate aminotransferase (IU/L)		26			
Alanine aminotransferase (IU/L)		14			
Alkaline phosphatase (IU/L)		227			
Gamma-glutamyl transferase (IU/L)		15			
Urinary hemoglobin		++			+
Urinary blood (red blood cells/microscopic field)		> 50			15

Reference values: Hemoglobin: 13-16 g/dL; Hematocrit: 40-54%; Leucocytes: 4,000-10,000/mm³; Platelets: 130,000-400,000/mm³; Lee-White Clotting test: Clottable; Urea: 10-45 mg/dL; Creatinine: 0.5-1.2 mg/dL, for adults; Aspartate aminotransferase: 6-34 IU/L in males; Alanine aminotransferase: 4-36 IU/L; Alkaline phosphatase: 20-140 IU/L; Gamma-glutamyl transferase: 7-60 IU/L, in males; Urinary hemoglobin: Negative; Urinary blood: no red blood cells/microscopic field.

This case report was approved by the ethics committee of the Universidade do Estado do Amazonas (CAAE 44853521.0.0000.5016; approval number 4.656.377/2021). The patient signed a consent form.

## DISCUSSION

In the Amazon region, there is a notable variety of vipers, though *Bothrops* is the most common genus associated with human envenomations. Nonetheless, *Lachesis* snakes cannot be ignored as they are also responsible for snakebite envenomations in this region. *L. muta* is a nocturnal, terrestrial, venomous pit viper found in areas of primary forest in South America and on the Caribbean Island of Trinidad^1^.

Our patient presented local symptoms of pain, swelling, and coagulation disturbance. Hypotension, hypothermia, and sudoresis were initially attributed to vagal stimulation caused by stress resulting from the snakebite episode. As *Bothrops* is by far the most frequently implicated animal, and symptoms were compatible, the patient initially received *Bothrops* antivenom, and it was considered to be a mild case. 

Consumption coagulopathy resulting in hypofibrinogenemia is also an important clinical consequence following envenoming by *Bothrops* and *Lachesis*. The venom of *L. muta* contains both a metalloproteinase fibrinogenase and a serine protease thrombin-like enzyme^9^, and the resulting coagulation disturbance is not neutralized by *Bothrops* antivenom. However, monitoring coagulation status is recommended after antivenom treatment, and normalization is expected to occur within 12-24 hours if the specific antivenom at the correct dosage is administered^10^. 

*Bothrops* and *Lachesis* venoms can cause indistinguishable local effects due to tissue damage at the site where the venom is introduced^11^. However, envenomations by *Lachesis* species are described to be more severe and are characterized by intense local edema, hemorrhage, and necrosis. 

Typical systemic manifestations of *Lachesis* envenomation include nausea, vomiting, diarrhea, bradycardia, and hypotension^8^ ^,^ ^10^. When present, such symptoms may indicate a differential diagnosis, and *Bothrops* envenomation should not be automatically assumed. In the present case, sudoresis and hypotension were not recognized as clinical effects of a *Lachesis* envenomation, although such symptomatology has been described previously^7^ ^,^ ^11^. Parasympathetic stimulation is not always described in envenomations in humans^12^. Differences in pharmacological, biochemical, or enzymatic characteristics of *Lachesis* venoms from different species and subspecies have not yet been detected^11^, and factors associated with vagal manifestations remain uncertain.

Laboratory parameters did not reveal thrombocytopenia, which has already been described in *L. muta* envenomations^7^ ^,^ ^8^, and thrombocytopenia is also observed in *B. atrox* envenomations^6^ ^,^ ^10^. Persistence of unclottable blood was the notable finding in this case and may occur for up to 15 days^6^. Additional vials of *Bothrops* antivenom did not alter the progression of the envenomation until the third day of hospital admission when *Lachesis* antivenom was finally administered, and coagulopathy was reversed.

In the present case, clinical diagnosis was not correctly made, although some symptoms that are suggestive of *Lachesis* envenomation were present upon admission to the hospital. Furthermore, the patient referred to the causative agent as “pico-de-jaca,” which is the popular name for *Lachesis* snakes, as opposed to “surucucu” for adult specimens of *B. atrox* ^1^. This aspect should be taken into account in the state of Acre and other parts of the Brazilian Amazon, and may help health professionals to establish the diagnosis, as few patients capture the causative agent of the snakebite^6^. 

This case report corresponds to the first reported *Lachesis* envenomation described in the state of Acre. The persistence of unclottable blood resulted from the lack of correct diagnosis. Consequently, the incorrect antivenom treatment during the first 3 days of the patient’s stay in the hospital, although characteristic symptoms of *Lachesis* envenomation were present upon admission to the hospital. Health professionals should be better trained to provide the correct treatment of snakebite via a diagnosis based on epidemiological information and regional aspects for the correct recognition of the causative snake.
